# A missense variant in *NCF1* is associated with susceptibility to unexplained recurrent spontaneous abortion

**DOI:** 10.1515/biol-2022-0518

**Published:** 2022-11-14

**Authors:** Mengxuan Du, Heng Gu, Yanqiu Li, Liyan Huang, Mengge Gao, Hang Xu, Huaqian Deng, Wenyao Zhong, Xiaohua Liu, Xingming Zhong

**Affiliations:** NHC Key Laboratory of Male Reproduction and Genetics, Guangdong Provincial Reproductive Science Institute (Guangdong Provincial Fertility Hospital), Guangzhou 510600, Guangdong Province, China; Department of Public Health and Preventive Medicine, School of Medicine, Jinan University, Guangzhou 510630, Guangdong Province, China

**Keywords:** NCF1, unexplained recurrent spontaneous abortion, reactive oxygen species, RELA

## Abstract

Unexplained recurrent spontaneous abortion (URSA) is a major concern in reproductive medicine. Neutrophil cytosolic factor 1 (*NCF1*) polymorphisms leading to low production of reactive oxygen species (ROS) are strongly associated with autoimmune diseases. We investigated the association of the missense single nucleotide polymorphism (SNP) rs201802880 (NCF1-339) in *NCF1* with URSA and explored its function. We performed NCF1-339 SNP genotyping of samples from 152 Chinese patients with URSA and 72 healthy controls using nested PCR and TaqMan assays. ROS production and RELA (NF-κB subunit) expression in the blood of participants with different NCF1-339 genotypes were determined. The frequencies of the wild-type (GG) and mutant (GA) genotypes remarkably differed between the URSA and control groups. The mutant genotype was associated with an increased risk of recurrent abortion. Furthermore, ROS levels in the URSA group with the GG genotype were significantly higher than those in the group with the GA genotype (*p* < 0.05). RELA expression in URSA patients with the GA genotype was considerably higher than that in control individuals with the GG genotype. These findings indicate that mutations in *NCF1* may increase the risk of URSA via the NADP/ROS/NF-κB signaling pathway, which has implications for the diagnosis and treatment of URSA.

## 1 Introduction

Recurrent spontaneous abortion (RSA), one of the most common complications of pregnancy, refers to having two or more consecutive spontaneous abortions with the same partner [1]. It occurs in up to 5% of women of reproductive age [2] and significantly impacts their physical and mental health. Moreover, epidemiological studies have found that pregnancies after RSA increase the risk of pregnancy-related complications, such as preeclampsia, fetal growth restriction, preterm birth, and stillbirth [3,4]. The etiology of RSA is complex. Factors known to lead to RSA include abnormal chromosome karyotypes of parents or embryos, abnormal uterine anatomy, endocrine and metabolic abnormalities, infectious diseases, antiphospholipid syndrome, and environmental factors [4,5]. However, approximately 50% of patients with RSA have unexplained RSA (URSA) with an unknown etiology [6].

Previous studies have shown that abortion results from the immunological rejection of embryos by pregnant women, leading to the failure of embryonic allotransplantation [7]. Some studies have suggested that pregnancy is a state of oxidative stress caused by enhanced activity of placental mitochondria and increased reactive oxygen species (ROS) levels [8]. Under normal circumstances, the embryo can cope with oxidative stress. However, when the ROS levels in the antioxidant system increase or decrease and there is an imbalance between free radical production and endogenous antioxidant defense mechanisms, the embryo is damaged in an inappropriate anoxic environment, leading to abortion [9].

In the human genome, neutrophil cytosolic factor 1 (NCF1) is located in the complex 7q11.23 region and encodes the p47phox/Ncf1 protein of the nicotinamide adenine dinucleotide phosphate (NADPH) oxidase (NOX2) complex, which is critical for ROS induction [10]. ROS can activate NF-κB, a major redox-regulated transcription factor, in response to inflammatory agonists [11]. The post-translational modification of RELA, a subunit of NF-κB, regulates NF-κB transcriptional activation and plays an important role in inflammation and inflammation-related diseases [12,13]. Recent studies have shown that the NF-κB signaling pathway can regulate maternal trophoblast differentiation, thereby playing a role in unexplained recurrent miscarriages [14]. The association of the NF-κB pathway with recurrent miscarriage was also supported by another study, conducted in 2019, targeting the relationship between the oxidative stress response and recurrent miscarriage [15].

Independently, many groups worldwide have associated the non-synonymous single nucleotide polymorphism (SNP) NCF1-339 (rs201802880) with multiple autoimmune diseases, such as systemic lupus erythematosus (SLE), chronic granulomatous disease (CGD), and rheumatoid arthritis [16–18]. However, no reports exist on the relationship between NCF1 mutations and unexplained recurrent miscarriage. To investigate the possible mechanisms underlying the role of ROS in URSA and the association between the NCF1-339 genotype and URSA, we studied the effects of the NCF1-339 genotype on ROS levels and RELA expression in patients with URSA. Our findings showed that NCF1 mutations may lead to URSA through the NADP/ROS/NF-κB signaling pathway, which has implications for URSA diagnosis and treatment.

## 2 Methods

### 2.1 Study populations

Case group (URSA group): A total of 152 patients with URSA, admitted to the Guangdong Reproductive Hospital between August 2019 and November 2021, were selected as the case group. Inclusion criteria were as follows: (1) two or more consecutive spontaneous abortions with a gestational age of <12 weeks; (2) normal maternal and paternal karyotypes; (3) absence of reproductive tract malformation, reproductive tract infection, TORTCH(+); (4) regular menstrual cycle, normal basic sex hormones, thyroid function, and fasting blood glucose levels; (5) absence of positive autoantibodies (antinuclear antibodies, anti-thyroid autoantibodies, anti-phospholipid antibodies); and (6) male semen routine examination.

Control group: A total of 72 normal women who underwent a medical examination during the same period were selected as the control group. The inclusion criteria were as follows: (1) previous normal birth history (one or more births); (2) no adverse pregnancy history, such as spontaneous abortion, stillbirth, and premature delivery; (3) no history of gestational diabetes, preeclampsia, or other complications of pregnancy, and a regular menstrual cycle; (4) no history or family history of autoimmune and metabolic diseases.

Peripheral blood samples (10 mL) were collected in the morning during non-pregnancy and non-menstrual periods after at least 8 h of fasting. A total of 5 mL of blood was added to anticoagulant tubes, containing 2% ethylenediaminetetraacetic acid, and stored at −80°C. The rest (5 mL blood) was centrifuged at 1,760×*g* for 10–15 min, and the separated serum was stored in tubes at −80°C.


**Informed consent:** Informed consent has been obtained from all individuals included in this study.
**Ethical approval:** The research related to human use has been complied with all the relevant national regulations, institutional policies and in accordance with the tenets of the Helsinki Declaration, and the protocol was approved by the Ethics Committee of Guangdong Institute of Reproductive Sciences (Guangdong Reproductive Hospital, 2021 12).

### 2.2 DNA and RNA extraction

A high-efficiency blood total RNA extraction kit was used to extract RNA (TIANGEN, CAT. No. DP443), and a DNA extraction kit was used to extract DNA (TIANGEN Cat. No. Dp304-02). DNA and RNA concentrations and the optical density values were determined using an ultra-micro-nucleic acid detector (Thermo Fisher, USA).

### 2.3 Whole exome sequencing (WES)

Whole exome capture: The Illumina TruSeq Exome Enrichment kit was used to capture the whole exome in the peripheral blood DNA of patients. WES: The IllunimaHiseq2500 sequencing platform was used for double-terminal WES of the captured sequences. Recognition of full exon group variation: The best practice recommended by GATK was used to identify SNPs and indels in the entire exon region. Compared with the 1000 Genomes Project data, mutation loci with a distribution frequency of less than 0.01 in the 1000 Genomes Project database were reserved. Four biological function prediction software packages (Mutation Taster, CADD, SIFT, and Polyphen2) were used [19–22].

### 2.4 Genotyping

To obtain the correct genotypes for the NCF1 variants, we specifically amplified the *NCF1* sequence using nested PCR and TaqMan assays. For more details, refer to Supplementary Material [23].

### 2.5 GO and KEGG enrichment analysis

GO enrichment analysis of variant genes was implemented using the clusterProfiler R package, in which gene length bias was corrected. Enrichment was considered significant when the corrected *p*-values were less than 0.05.

### 2.6 ROS production

ELISA kits (JINGMEI, Cat. No. JM-0601H1) were used to detect the concentrations of cytometric factors (ROS) using the double antibody sandwich method. Samples, standard substances, and HRP-labeled detection antibodies were added to the ROS-coated micropores once. Following the incubation, samples were thoroughly washed. The substrate tetramethylbenzidine (TMB) was used for color rendering. TMB was converted to blue under the catalysis of peroxidase and yellow under the action of acid. Color intensity was positively correlated with ROS levels in the sample. We measured the absorbance (OD value) using an enzyme-labeled microplate reader (TECAN, Switzerland) at a wavelength of 450 nm to calculate the concentration using a standard curve.

### 2.7 Gene expression of *RELA*

The experiment was performed following the instructions in the reverse transcription kit (Cat. No. RR036A). mRNA expression of RELA was measured using the TB Green Premix Ex Taq II Kit (TAKARA, Cat. No. RR820A) in a StepOnePlus™ Real-Time PCR System with a Tower (ABI, USA). Three assays were performed per sample. Data were analyzed using the 2^−ΔΔCt^ method, with β-actin as the internal control. The primers used for the qRT-PCR are listed in Table 1.

**Table 1:** Primers for RELA gene using reverse transcription polymerase chain reaction

Gene	Position	Primer	Seq
RELA	NM_001145138.2	RELA-F	GCGAGAGGAGCACAGATACC
		RELA-R	CTGATAGCCTGCTCCAGGTC

### 2.8 Statistical analysis

The SPSS.26 software was used for data analysis, and GraphPad Prism6 was used for graph construction. The chi-square test was used for comparative analysis of genotypes, and logistic regression analysis was used to calculate the odds ratio (OR) values and 95% confidence intervals (CI) to evaluate the relationship between NCF1 variation and URSA susceptibility. Gene expression, ELISA data, and data grouping were compared, and normality tests were conducted. The homogeneity of variance test was performed for normally distributed data. The *t*-test was used for data comparison between the two groups, and the rank test was used for data that did not conform to a normal distribution. Statistical significance was set at a *p*-value of <0.05.

## 3 Results

### 3.1 WES

**Figure 1:**
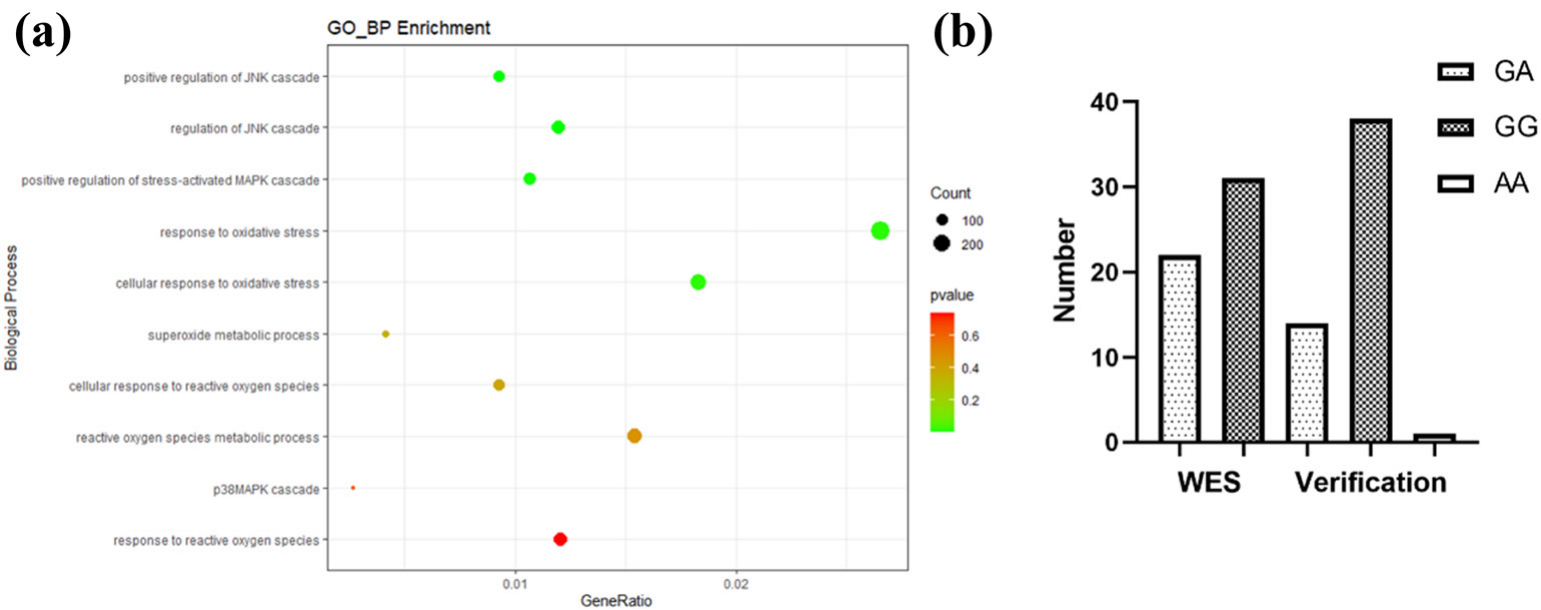
Gene ontology of mutated genes and NCF1 sequence diagram: (a) NCF1 is involved in biological processes related to oxidative stress and (b) verification results.

After WES of the samples obtained from the 53 patients with URSA, function prediction was carried out using Mutation Taster, CADD, SIFT, and Polyphen2. Compared with the 1000 Genome Project data, non-benign genes with low mutation frequencies were screened. GO enrichment analysis of the selected genes (Figure 1a) revealed that NCF1 was enriched in biological processes such as oxidative stress and immunity. Furthermore, WES showed that *NCF1* had a high mutation frequency in this patient sample (22/53). When 53 samples were verified, it was found that only 15 of the 22 variants had *Ncf1* variants, one of which was pure and mutant (Figure 1b).

### 3.2 Demographic data and genotyping association analyses

In addition to the 53 patients with URSA initially included in the WES analysis, we recruited another 99 patients with URSA. The final sample sizes of the patient and healthy control groups were 152 and 72, respectively. The basic demographic data of the URSA and control groups are shown in Table 2. There was no statistically significant difference in age and BMI between the two groups (*p* > 0.05). Subsequently, the different genotypes of NCF1-339 were analyzed and compared; the results are shown in Table 3. The frequencies of the wild-type (GG) and mutant (GA) genotypes were significantly different between the two groups (*p* < 0.05). The GA mutant was significantly associated with the risk of recurrent abortion (GA vs GG: OR = 3.257, 95% CI: 1.494–7.102). There were only four homozygous mutations (AA) in the URSA group, but the differences were not statistically significant because of the small number of controls included.

**Table 2:** Comparison of basic data between case group and control group

	Control (*n* = 72)		URSA (*n* = 152)		*p*-value
	Mean	SD	Mean	SD	
AGE	28.75	4.35	29.14	3.71	0.483
BMI	21.26	1.34	21.29	1.36	0.892

Data are shown as mean ± SD; BMI, body mass index; *p*-value < 0.05 was considered as significant.

**Table 3:** Genotype and allele frequencies of the NCF1 gene in URSA patients and controls

	Control (*n* = 72)	URSA (*n* = 152)	*χ* ^2^	OR (95% CI)**
GG	63	101	9.46*	1
GA	9	47	—	3.257 (1.494–7.102)
AA	—	4	—	—
G	135	249	11.19*	
A	9	55		3.313 (1.588–6.911)

^*^Distribution of genotypes between URSA group and control group was detected by *χ* ^2^ test, *p* < 0.05; **OR value and 95% CI were calculated by logistic regression.

### 3.3 GA genotype of NCF1-339 reduces extracellular ROS production

**Figure 2:**
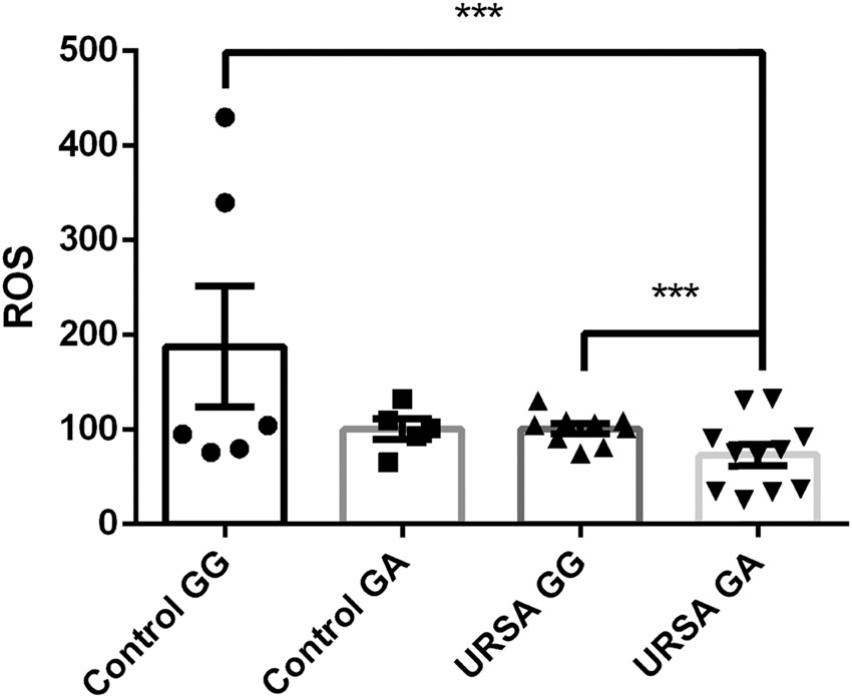
ELISA results. ROS levels were expressed in each group.

To determine if the GA genotype of NCF1-339 affects ROS production, we excluded factors that may affect ROS production, including hormones, pregnancy, abortion, and drugs. We used ELISA to detect the production of extracellular ROS in the URSA (GG, GA) and control (GG, GA) groups. After group comparisons, we found that ROS levels in the URSA group individuals with the GG genotype were significantly higher than those in the URSA group individuals with the GA genotype, and the difference was statistically significant (GG vs GA: 101.20 ± 15.72 vs 73.05 ± 35.82, *p* < 0.05). Thus, the GA genotype of NCF1-339 reduced the ROS levels (Figure 2). Although not statistically significant, the ROS levels of individuals in the control group with the GA genotype were lower than those of individuals with the GG genotype.

### 3.4 GA genotype of NCF1-339 increases the expression of RELA

**Figure 3:**
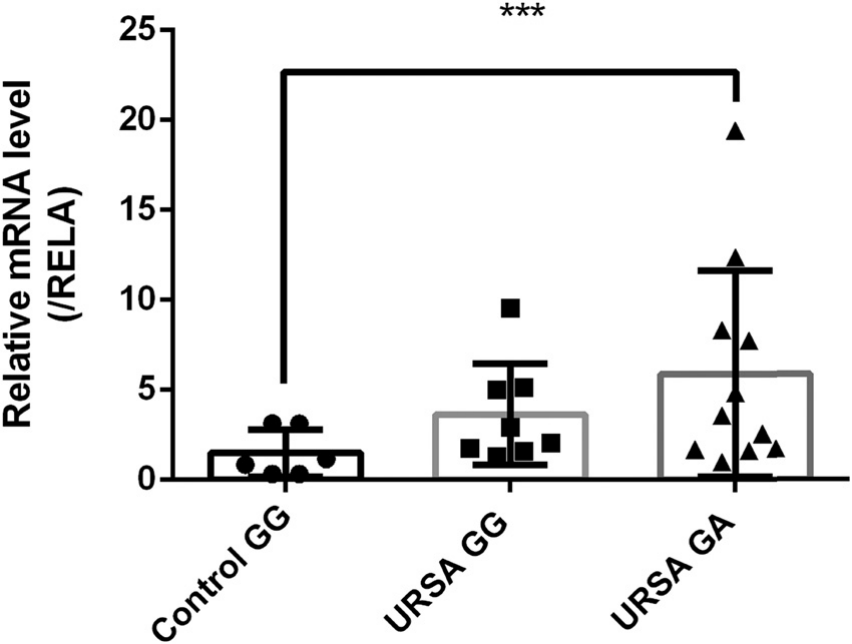
Relative mRNA level (RELA). ***p < 0.05.

After detecting ROS levels using ELISA, we further explored the NF-κB signaling pathway downstream of ROS related to inflammatory immunity. We used qRT-PCR to detect the expression of *RELA*. The expression of *RELA* in URSA patients with the GA genotype was significantly higher than that in control individuals with the GG genotype (*p* < 0.05). In addition, the GA genotype had higher ROS levels than the GG genotype in the URSA group, which may be due to the smaller number of samples included; therefore, the difference was not significant (Figure 3). After group comparisons, we found that a reduction in ROS levels weakened their inhibitory effect on RELA, subsequently increasing RELA expression.

## 4 Discussion

The unknown etiology of URSA makes diagnosis and treatment of patients difficult. Studies suggest that immune dysfunction may play an important role in the pathogenesis of URSA. WES technology is increasingly being used in scientific research and clinical areas [1]. The genetic diagnosis of human diseases has improved remarkably through WES technology, especially for non-related patients. Different WES strategies have been used to diagnose reproductive disorders [2–4], which has increased the understanding of gene function and interactions in human reproduction [5]. The WES technique with bioinformatic analysis allows the mining of genetic information about mutations in risk factors leading to pregnancy loss. Thus, the detection of peripheral blood from URSA patients via WES technology to screen for genetic variants associated with immune regulation may help to find the potential cause of the disorder and provide new strategies for its diagnosis and treatment. In this study, whole-exome sequencing analysis revealed possible NCF1 high-frequency variants with enriched functions primarily focused on immunity.

The NCF1 gene encodes the protein NCF1, also known as p47phox, a component of NOX2. NOXs can reduce oxygen molecules in the body to superoxide negatives via the reduced NADPH-dependent one electron reduction, and this family is a major source of ROS [24]. ROS generated catalytically by NOX plays important roles in many physiological processes, notably immune defense. Current studies of the regulation of autoimmune inflammatory genes mainly focus on NCF1 and induced ROS function. In humans, deleterious variants in genes encoding proteins of the NOX2 complex lead to ROS deficiency and the development of CGD [25]. Zhao et al. identified the variants in NCF1 associated with SLE and found that reduced NOX2 mediated the changes in ROS levels, playing a role in autoimmune diseases [26]. Variations in human NCF1, including copy number variations and SNPs, result in impaired immunity, as the enzyme is unable to function as an NADPH oxidase to trigger the oxidative burst in phagocytes, inducing autoimmune diseases such as arthritis, autoimmune lung disease, and lupus erythematosus. Genome-wide association studies (GWAS) have identified dozens of genetic loci associated with SLE, identifying a common variant in NCF1, c.269g>A,p. The r90h (rs201802880) variant is a novel risk variant for SLE, Sjogren’s syndrome, and rheumatoid arthritis [27].

The variant site of NCF1 c.269G > A,p. R90H (rs201802880) is located in the active site of NAPDH oxidase in the hot spot mutation region. Of note, there are two pseudogenes close to NCF1: NCF1B and NCF1C (NCF1B/C), which encode truncated, non-functional proteins sharing 98% sequence similarity with NCF1 [28]. This complicates genotyping and excludes NCF1 from GWAS. NCF1 variants have not been correctly identified in studies using short sequence reads, such as the 1000 Genomes Project and Exome Aggregation Consortium, owing to the presence of NCF1B and NCF1C. In this study, to obtain the correct genotypes for NCF1 variants, we specifically verified the NCF1 sequences of 22 individuals in which heterozygous variation was detected using WES, nested PCR, and TaqMan assays. The 22 samples were validated, and only 15 samples were found to have mutations in NCF1.

Independent studies confirm a strong association of the non-synonymous SNP NCF1-339 (rs201802880) with multiple autoimmune diseases, such as SLE, CGD, and rheumatoid arthritis [29–31]. This shows that NCF1-339 is one of the strongest SNPs outside the human leukocyte antigen region that is associated with autoimmune diseases. We report that an amino acid replacement (NCF1-339, Arg90His) in NCF1, which leads to a lower capacity to induce oxidative burst, is strongly associated with URSA. The OR was 3.257, making it one of the strongest identified genetic associations with URSA.

The NCF1-339 A allele leads to a shift from Arg to His at position 90, located in the phox domain of NCF1, which mediates its binding to the cell membrane. The substitution of evolutionarily conserved Arg90 with an encoded histidine residue was predicted to be deleterious. We, and others, have previously shown that mutation at position 90 reduces the ROS response [26] and the binding efficiency of NCF1 to the membrane. ROS can be a double-edged sword in the field of autoimmunity. High levels of ROS, predominantly produced by NOX2 in phagocytes for host defense, may lead to inflammatory tissue damage. However, ROS are also signaling molecules that regulate T cell differentiation, B cell proliferation, and antigen processing in dendritic cells [26]. Studies report that decreased ROS production can increase the risk of developing autoimmune diseases [16,32–35]. Our findings suggest that p. Arg90His might confer a risk for URSA by reducing the NOX2-derived ROS levels.

ROS have numerous downstream signaling factors, among which NF-κB is a dominant transcription factor in the cellular inflammatory response directly involved in the inflammatory response of tissues, while inducing multiple proinflammatory cytokines, including TNF, and the transcription of IL-1 and IL-6, playing a key role in the autoimmune disease process [36,37]. In this study, we found that decreased ROS levels lead to elevated expression of the RELA gene (NF-κB subunit), which is likely to be because ROS are involved in regulating the initiation of the NF-κB pathway. A literature search shows that ROS are able to promote the NF-κB signaling pathway via IκBα Tyrosine phosphorylation (Tyr42) and pset domain phosphorylation, which induce NF-κB p65 (Ser529) phosphorylation and activation. Consistent with our findings, the aberrant ROS levels in SLE triggered the activation of transcription factors, such as NF-κB and AP-1, which led to the excessive secretion of proinflammatory factors, resulting in the weakening of the antioxidant defense system and oxidative stress [38].

In summary, we identified a p.Arg90His substitution in NCF1 (NCF1-339, rs201802880) as a novel risk variant for URSA. NCF1 gene variation may lead to URSA through the NADPH/ROS/NF-kB signaling pathway, which is of great clinical significance. However, this is only a preliminary association study, and further research is required into the specific mechanisms by which the NCF1-339 variant leads to maternal autoimmune disorders and immune imbalances at the maternal–fetal interface, to confirm the association with miscarriage.

## Supplementary Material

Supplementary Material
