# Supplementary material for: A missense variant in NCF1 is associated with susceptibility to unexplained recurrent spontaneous abortion
Source: Open Life Sci. 2022 Nov 14;17(1):1443–50. doi: 10.1515/biol-2022-0518 (PMC9663937; doi:10.1515/biol-2022-0518)
Supplement: Supplementary Material [file biol-2022-0518-sm.pdf]

Supplementary material

S1 Genotyping of target loci by nested PCR combined with TaqMan fluorescent probe method

Nested PCR reaction system: Take a PCR tube, number it with a marker, and prepare it according to the method provided by the PCR detection kit. Take 5 µL of TaKaRa Taq HS Perfect mix, 1 µL of nested PCR primer Mix, and 4 µL of DNA template + ddH_2O. The total reaction volume was 10 µL.

Nested PCR reaction conditions: place the prepared reaction system in a PCR amplicon, and set the conditions as follows: A: pre-denaturation at 94°C for 5 minutes, B: cyclic reaction at 94°C for 30 s; 60°C for 30 s; 68°C 1 min; 34 cycles total; 72°C 7 min, C: store 4°C.

Electrophoresis: After the amplification reaction was terminated, the reaction products were detected by 1.2% agarose gel electrophoresis. Put the prepared 1.2% agarose gel into the electrophoresis tank, pour enough 1× TAE electrophoresis buffer to cover the agarose gel, take 5 µL of the amplified product, and give 100 V constant pressure electrophoresis for 30 min. Take pictures on a gel imager and observe the results.

Real-time quantitative polymerase chain reaction (RT-PCR) + TaqMan fluorescent probe genotyping: RT-PCR was used to amplify the target fragment and TaqMan fluorescent probe technology was used to detect the NCF1 locus rs201802880 to detect genotyping.

Real-time quantitative PCR reaction system: Dilute the product amplified by nested PCR by 100 times and use it as a DNA template. The total volume of the reaction system is 20 µL, including 2 µL of DNA template, 10 µL of Probe qPCR Mix (2x), and 0.8 µL of real-time quantitative PCR primer mix. ROX Reference Dye 0.4 µL, Probe Mix 1 µL, ddH2O 5.8 to 20 µL. Real-time quantitative PCR reaction conditions: After short centrifugation of the prepared reaction system, place it in an ABI real-time fluorescence quantitative PCR instrument for amplification. The conditions are as follows: A: Pre-denaturation at 95°C for 20 s, B: Cyclic reaction at 95°C 1 s; 60°C 20 s; 40 cycles total, 60°C 30 s. After the reaction, the end-point fluorescence in the sample well was read on the ABI real-time fluorescence quantitative nucleic acid amplifier, and the different fluorescence was converted into DNA sequence through the analysis software, so as to interpret the genotype of each sample and record it.

S2 Enzyme-linked immunosorbent assay

1. Remove the required slats from the aluminum foil bag after equilibrating at room temperature for 20 min, and seal the remaining slats with a ziplock bag and return to 4°C.
2. Set standard wells and sample wells, and add 50 µL of different concentrations of standard to each standard well.
3. First add 10 µL of the sample to be tested to the sample well, then add 40 µL of sample diluent; do not add to the blank.
4. Add 100 µL of HPR-labeled antibody to be tested to each well of the standard wells and sample wells except for the blank wells, seal the reaction wells with a sealing film, and incubate in a 37°C water bath or incubator for 60 min.
5. Discard the liquid, pat dry on absorbent paper, fill each well with washing liquid, let stand for 1 min, shake off the washing liquid, pat dry on absorbent paper, and repeat the plate washing 5 times.
6. Add 50 µL of substrates A and B to each well, and incubate at 37°C for 15 min in the dark.
7. Add 50 µL of stop solution to each well, and measure the OD value of each well at a wavelength of 450 nm within 15 min.
8. Draw the standard curve: In the Excel worksheet, take the standard concentration as the abscissa and the corresponding OD value as the ordinate, draw the standard linear regression curve, and calculate the concentration value of each sample according to the curve equation.
